# Supplementary material for: FERN – a Java framework for stochastic simulation and evaluation of reaction networks
Source: BMC Bioinformatics. 2008 Aug 29;9:356. doi: 10.1186/1471-2105-9-356 (PMC2553347; doi:10.1186/1471-2105-9-356)
Supplement: Additional file 1 — FERN distribution, Version 1.3. This archive contains the FERN source code and binaries as well as documentation and example models in FernML and SBML. [file 1471-2105-9-356-S1.zip › fern/doc/javadoc/fern/simulation/observer/RandomNumberGeneratorCallObserver.html]

RandomNumberGeneratorCallObserver


---


|  |  |  |
| --- | --- | --- |
| **Overview** \| **Package** \| **Class** \| **Use** \| **Tree** \| **Deprecated** \| **Index** \| **Help** | |  |
| **PREV CLASS**   **NEXT CLASS** | **FRAMES**    **NO FRAMES**     **All Classes** |  |
| SUMMARY: NESTED \| FIELD \| CONSTR \| METHOD | DETAIL: FIELD \| CONSTR \| METHOD |  |


---


## fern.simulation.observer Class RandomNumberGeneratorCallObserver

```
java.lang.Object
  fern.simulation.observer.Observer
      fern.simulation.observer.RandomNumberGeneratorCallObserver
```

**All Implemented Interfaces:**: GnuPlotObserver

---

``` public class RandomNumberGeneratorCallObserver extends Observer implements GnuPlotObserver ```

Observes the calls to the random number generator and count the number of drawn
random number of different distributions. These calls are crucial for the stochastic
algorithms because besides algebraic calculations are these the most time
consuming operations.

This observer does take repeats into account. If you repeat the simulation,
you will get an average over the results each run.

**Author:**
:   Florian Erhard

**See Also:**: `Stochastics`

---

**Constructor Summary**

`RandomNumberGeneratorCallObserver(Simulator sim)`
  
          Creates the observer for the given simulator.


| **Method Summary** | |
| --- | --- |
| `void` | `activateReaction(int mu, double tau, Simulator.FireType fireType, int times)`             Do nothing. |
| `void` | `finished()`             Calculates the average. |
| `String[]` | `getStyles()`             Gets the styles for the columns. |
| `void` | `started()`             Resets the count variables of the random number generator calls. |
| `void` | `step()`             Do nothing. |
| `void` | `theta(double theta)`             Do nothing. |
| `GnuPlot` | `toGnuplot()`             Creates a new `GnuPlot` object and passes the actual observer data to it. |
| `GnuPlot` | `toGnuplot(GnuPlot gnuplot)`             Passes the actual observer data to a `GnuPlot` object. |
| `String` | `toString()` |

**Methods inherited from class fern.simulation.observer.Observer**

`applyLabelFormat, getLabel, getLabelFormat, getNumSimulations, getPrintWriter, getSimulator, getTheta, getTitlesCommand, print, setLabelFormat, setPrintWriter, setSimulator, setTheta`

**Methods inherited from class java.lang.Object**

`clone, equals, finalize, getClass, hashCode, notify, notifyAll, wait, wait, wait`

**Constructor Detail**

### RandomNumberGeneratorCallObserver

```
public RandomNumberGeneratorCallObserver(Simulator sim)
```

:   Creates the observer for the given simulator.

    **Parameters:**: `sim` - simulator


**Method Detail**

### activateReaction

```
public void activateReaction(int mu,
                             double tau,
                             Simulator.FireType fireType,
                             int times)
```

:   Do nothing.

    :   **Specified by:**: `activateReaction` in class `Observer`
    :   **Parameters:**: `mu` - the reaction which is supposed to fire: `tau` - the time the reaction fires (at this time `Simulator.getTime()` does not necessarily yield the firing time): `fireType` - the type of the firing: `times` - TODO

---


### finished

```
public void finished()
```

:   Calculates the average.

    :   **Specified by:**: `finished` in class `Observer`

---


### started

```
public void started()
```

:   Resets the count variables of the random number generator calls.

    :   **Specified by:**: `started` in class `Observer`
    :   **See Also:**: `Stochastics.resetCounts()`

---


### step

```
public void step()
```

:   Do nothing.

    :   **Specified by:**: `step` in class `Observer`

---


### theta

```
public void theta(double theta)
```

:   Do nothing.

    :   **Specified by:**: `theta` in class `Observer`
    :   **Parameters:**: `theta` - moment in time

---


### getStyles

```
public String[] getStyles()
```

:   **Description copied from interface: `GnuPlotObserver`**
:   Gets the styles for the columns. If you don't want styles, just return null!

    :   **Specified by:**: `getStyles` in interface `GnuPlotObserver`
    :   **Returns:**: styles for the columns

---


### toGnuplot

```
public GnuPlot toGnuplot()
```

:   **Description copied from interface: `GnuPlotObserver`**
:   Creates a new `GnuPlot` object and passes the actual observer data to it.

    :   **Specified by:**: `toGnuplot` in interface `GnuPlotObserver`
    :   **Returns:**: the created `GnuPlot` object

---


### toGnuplot

```
public GnuPlot toGnuplot(GnuPlot gnuplot)
```

:   **Description copied from interface: `GnuPlotObserver`**
:   Passes the actual observer data to a `GnuPlot` object.

    :   **Specified by:**: `toGnuplot` in interface `GnuPlotObserver`
    :   **Parameters:**: `gnuplot` - the `GnuPlot` object to pass the data to **Returns:**: the `GnuPlot` object

---


### toString

```
public String toString()
```

:   **Overrides:**: `toString` in class `Object`


---


|  |  |  |
| --- | --- | --- |
| **Overview** \| **Package** \| **Class** \| **Use** \| **Tree** \| **Deprecated** \| **Index** \| **Help** | |  |
| **PREV CLASS**   **NEXT CLASS** | **FRAMES**    **NO FRAMES**     **All Classes** |  |
| SUMMARY: NESTED \| FIELD \| CONSTR \| METHOD | DETAIL: FIELD \| CONSTR \| METHOD |  |


---
